# Supplementary material for: Coping strategies and psychological distress among mothers during COVID-19 pandemic: The moderating role of social support
Source: PLoS One. 2024 Apr 2;19(4):e0300365. doi: 10.1371/journal.pone.0300365 (PMC10986941; doi:10.1371/journal.pone.0300365)
Supplement: S1 File — (PDF) [file pone.0300365.s002.pdf]

KEMENTERIAN PENDIDIKAN DAN KEBUDAYAAN
UNIVERSITAS PADJADJARAN
KOMISI ETIK PENELITIAN
RESEARCH ETHICS COMMITTEE
Jl. Prof. Eyckman No. 38 Bandung 40161
Telp. & Fax. 022-2038697 email: etik.unpad@gmail.com, website: kep.unpad.ac.id

No. Reg.: 0720040490

PERSETUJUAN ETIK
ETHICAL APPROVAL

Nomor: 486 /UN6.KEP/EC/2020

Komisi Etik Penelitian Universitas Padjadjaran Bandung, dalam upaya melindungi hak asasi dan kesejahteraan subjek penelitian serta menjamin bahwa penelitian yang menggunakan formulir survei/registrasi/surveilens/ Epidemiologi/Humaniora/Sosial Budaya/Bahan Biologi Tersimpan/Sel Punca dan non klinis lain nya berjalan dengan memperhatikan implikasi etik, hukum, sosial dan non klinis lainnya yang berlaku, telah mengkaji dengan teliti proposal penelitian berjudul:

The Research Ethics Committee Universitas Padjadjaran Bandung, in order to protect the rights and welfare of the research subject, and to guaranty that the research using survey questionnaire/registry/surveillance/ epidemiology/humaniora/social-cultural/archived biological materials/stem cell/other non clinical materials, will carried out according to ethical, legal, social implications and other applicable regulations, has been throughly reviewed the proposal entitled:

"GAMBARAN KESEHATAN MENTAL IBU PADA MASA PANDEMI COVID-19 DI INDONESIA"

Nama Peneliti Utama<br>*Principal Researcher*	:	Fredrick Dermawan Purba, Ph.D., Psikolog
Pembimbing/Peneliti Lain<br>*Supervisor/Other Researcher*	:	Fitri Ariyanti Abidin, S.Psi., M.Psi.<br>Fitriani Yustikasari Lubis, S.Psi., M.Psi.<br>Laila Qodariah, S.Psi., M.Psi., Psikolog<br>Vidya Anindhita, S.Psi., M.Psi.
Nama Institusi<br>*Institution*	:	Fakultas Psikologi<br>Universitas Padjadjaran

proposal tersebut dapat disetujui pelaksanaannya.
hereby declare that the proposal is approved.

Ditetapkan di : Bandung
Issued in
Tanggal : 15-05-2020
Date

Ketua,
Chairman,

Dr. Meita Dhamayanti, dr., SpAK., M.Kes
NIP. 196305191987122001

Keterangan/*notes*:
Persetujuan etik ini berlaku selama satu tahun sejak tanggal ditetapkan.
This ethical clearance is effective for one year from the due date.
Pada akhir penelitian, laporan pelaksanaan penelitian harus diserahkan ke Komisi Etik Penelitian.
In the end of the research, progress and final summary report should be submitted to the Research Ethics Committee.
Jika ada perubahan atau penyimpangan protokol dan/atau perpanjangan penelitian, harus mengajukan kembali permohonan kajian etik penelitian.
If there be any protocol modification or deviation and/or extension of the study, the Principal Investigator is required to resubmit the protocol for approval.
Jika ada kejadian serius yang tidak diinginkan (KTD) harus segera dilaporkan ke Komisi Etik Penelitian.
If there are Serious Adverse Events (SAE) should be immediately reported to the Research Ethics Committee
