# Supplementary material for: Coping strategies and psychological distress among mothers during COVID-19 pandemic: The moderating role of social support
Source: PLoS One. 2024 Apr 2;19(4):e0300365. doi: 10.1371/journal.pone.0300365 (PMC10986941; doi:10.1371/journal.pone.0300365)
Supplement: Attachment — Submitted filename: Response to Reviewers (1).docx [file pone.0300365.s003.docx]

Submission **Coping strategies and psychological distress among mothers during Covid-19 pandemic: the moderating role of social support**

Dear reviewer(s),

First, we would like to thank you for your constructive input and for recognizing our effort to improve this paper. Those comments genuinely boost our motivation to work better on our current study. Regarding your feedback, below we have written down your comments, which we put in *italics*. Your comments are followed by our response and the associated changes made in the manuscript (identified by track changes). You will find that we adapted the text in all cases. We hope that you will appreciate the new version, and we look forward to finalizing this draft and preparing it for publication.

**Reviewer #1**

**Overall comment**

1. **Comments :**

*Reviewer #1: The authors dealt with an important topic; the novelty is that they focused on coping strategies and psychological distress among mothers during the COVID-19 pandemic, with special emphasis on social support.*

**Our response:**

We thank the reviewer for the constructive comments on this manuscript.

2. **Comments :**

*Use past tense throughout the entire text, except for the description of measures, where you use the present tense.*

**Our response:**

We checked the whole manuscript and used past tense.

**Abstract**

3. **Comments :**

*Abstract – please report participants’ mean age.*

**Our response:**

We added the *participants’ mean age* and standard deviation

**Changes made in the text:**

Line 9: (Mean age 37.12 years; SD 6.63)

4. **Comments :**

*Abstract – “The result shows that social support moderates both in the relationship between approach coping strategy and psychological distress (B=.041, CI .007-.075) and
between avoidant coping strategy and psychological distress (B=-.100, CI -.138--.061).”*

*Try to being much more specific, such as: “The result shows that HIGHER/LOWER social support moderates both in the relationship between HIGHER/LOWER approach coping strategy and HIGHER/LOWER psychological distress (B=.041, CI .007-.075) and between avoidant HIGHER/LOWER coping strategy and HIGHER/LOWER psychological distress (B=-.100, CI -.138--.061).*

**Our response:**

We thank the reviewer for this comment.

**Changes made in the text:**

Line 12-18: The result shows that moderate and high level of social support moderates the relationship between approach coping strategy and psychological distress (B=.041, CI .007-.075). It means when mother used approach coping, her psychologycal distress mother will decreased further whenever she received moderate and high level of social support. Any level of social support moderates the relationship between avoidant coping and psychological distress (B=-.100, CI -.138--.061). It means when mother used avoidant coping, her social support at any level served as buffer to her psychological distress.

5. **Comments :**

*Abstract – “The difference lies in the conditional effect of social support.” This sentence appears erratically presented. Please improve.*

**Our response:**

Similar to the previous comment.

**Changes made in the text:**

Line 12-18: The result shows that moderate and high level of social support moderates the relationship between approach coping strategy and psychological distress (B=.041, CI .007-.075). It means when mother used approach coping, her psychologycal distress mother will decreased further whenever she received moderate and high level of social support. Any level of social support moderates the relationship between avoidant coping and psychological distress (B=-.100, CI -.138--.061). It means when mother used avoidant coping, her social support at any level served as buffer to her psychological distress.

6. **Comments :**

*Abstract – ““Social support moderates…effect”; this sentence is difficult to understand.*

**Our response:**

Similar to the previous comment.

**Changes made in the text:**

Line 12-18: The result shows that moderate and high level of social support moderates the relationship between approach coping strategy and psychological distress (B=.041, CI .007-.075). It means when mother used approach coping, her psychologycal distress mother will decreased further whenever she received moderate and high level of social support. Any level of social support moderates the relationship between avoidant coping and psychological distress (B=-.100, CI -.138--.061). It means when mother used avoidant coping, her social support at any level served as buffer to her psychological distress.

7. **Comments :**

*Abstract – “The results…. distress.” Please revise this part, as these assumptions are difficult to understand.*

**Our response:**

Similar to the previous comment.

**Changes made in the text:**

Line 12-18: The result shows that moderate and high level of social support moderates the relationship between approach coping strategy and psychological distress (B=.041, CI .007-.075). It means when mother used approach coping, her psychologycal distress mother will decreased further whenever she received moderate and high level of social support. Any level of social support moderates the relationship between avoidant coping and psychological distress (B=-.100, CI -.138--.061). It means when mother used avoidant coping, her social support at any level served as buffer to her psychological distress. .

**Main Manuscript**

8. **Comments :**

*Main Manuscript – Introduction: “… greatly impactED the mental….”; as mentioned above, use past tense throughout the text.*

**Our response:**

We checked the whole manuscript and used past tense.

9. **Comments :**

*Main Manuscript – Introduction: “Government responses such as lockdown and quarantine policies might stimulate stress, feeling of uncertainty, anxiety and psychological distress.”; please add references.*

**Our response:**

We added as suggested.

**Changes made in the text:**

Line 25-29: Government regulations such as lockdown and quarantine policies had an adverse effect on adults’ mental health around the world, as evidenced by an increase in depression and stress-related symptoms [2,3], increased parental burnout [4], and the level of parent’s stress have not yet returned to pre-COVID-19 levels [5].

10. **Comments :**

*Main Manuscript – Introduction: “Parents, among others, are also being affected significantly by this pandemic. Parents’ well-being in various aspects of their life: e.g., health, safety, economic might be threaten of these pandemic-related stressors.” Overall, I do kindly but strongly ask you to be much more specific with your claims: ““Parents, among others, are also being affected significantly by this pandemic” But why should this happen?. Parents’ well-being in various aspects of their life: e.g., health, safety, economic might be threaten of these pandemic-related stressors.”; but why should they be threatened? And all really all parents under stress? Did the COVID-19 pandemic negatively impact on all parents, individuals and adults?*

**Our response:**

We added relevant studies that show that almost everyone was negatively impacted by the Covid-19 Pandemic.

**Changes made in the text:**

Line 29-38: Among others, parents have experienced enormous stress by this pandemic. Due to disruptions in their lives at work, finance, children’s education and in their child care, parents had to deal with a great stressor. [6]. According to a previous study, parents experienced higher levels of stress in many areas of their lives, including work (e.g. unemployment, work-from-home arrangements), family (e.g inability to meet in person, managing children’s education), relationship (conflict with a spouse, lack of support), physical (e.g. poor sleep_, and psychological (e.g. anxiety, depression) [7]. Another previous study found that parental mental health issues are associated with low material assets in a pandemic (such as financial security, food security, and children having access to outdoor space), familial assets (such as parents’ time for themselves), and community assets (receiving support from others outside the household) [8]

11. **Comments :**

*Main Manuscript – Introduction: “Among these effects of the COVID-19 pandemic toward parenting, specific and more profound effects had been found in the maternal roles.” Please add references; why did the COVID-19 pandemic and its related restrictions impact more severely on mothers? Is it possible that there is a systematic gender-related study bias, such that fathers were not asked, when compared to mothers?*

**Our response:**

We added relevant literature that collects data not only from mothers but also from fathers: Bastiaansen et al. (2021). They found the relationship between state-imposed COVID-19 lockdown measures, co-parenting mitigation, and levels of parental burnout was different between parents: mitigating significant for fathers and non-significant for mothers. It proved the *COVID-19 pandemic impacted more severely on mothers.*

**Changes made in the text:**

Line 43-49 : Among these effects of the COVID-19 pandemic toward parenting, specific and more profound effects had been found in the maternal roles. These effects can be exacerbated in working mothers. According to a previous study, co-parenting had different effect on men and women in pandemic situation [4]. During the pandemic, working mothers spent more time in childrearing and homeschooling than working fathers [11]. The previous study reported that work arrangements during the pandemic resulted in unideal merger of work and personal space of working mothers [12].

12. **Comments :**

*Main Manuscript – Introduction: “…since March 2020.”; please add the time lapse.*

**Our response:**

Time-lapse was edit

**Changes made in the text:**

Line 51: since March 2020 until July 2021

13. **Comments :**

*Main Manuscript – Introduction: Social support; please introduce a subheading.*

**Our response:**

We changed the subheading.

**Changes made in the text:**

Line 76 : **Social Support**

14. **Comments :**

*Main Manuscript – Introduction: “…. performed by Khalid, A., & Dawood, S. (2020) also revealed… “please double-check referencing style.*

**Our response:**

Referencing style was *double-checked*.

**Changes made in the text:**

Line 89-91 : Social support, self-efficacy, active-practical coping, and active-distractive are negatively associated with psychological distress [30].

15. **Comments :**

*Main Manuscript – Introduction: Please formulate hypotheses and showcase in much more details, if and to what extent the present data add to the current literature in a new fashion.*

**Our response:**

We thank the reviewer for the constructive comments to improve this manuscript.

**Changes made in the text:**

-

16. **Comments :**

*Main Manuscript – Method: while this is often a question of taste and judgement, please start with the description of the participants, then mention the procedure, before you describe the measures.*

**Our response:**

As suggested, we moved the participants and procedure sections before the measures section.

17. **Comments :**

*Main Manuscript – Method: Participants: inclusion and exclusion criteria: does “aged more than 17 years” equal to “at least 18 years and older”?.*

**Our response:**

We revised it as suggested

**Changes made in the text:**

Line 146 : at least 18 years and older

18. **Comments 1 :**

*Main Manuscript – SPSS® 21.0 (IBM Corporation, Armonk NY, USA). As regards the use of non-parametric procedures, with such a large sample size, you may use parametric procedures. Please explain, if minimum criteria to run regression models were met.*

**Our response:**

We thank the reviewer for pointing out that we are able to use parametric procedures. We also added a sentence regarding minimum criteria to run regression models, as suggested.

**Changes made in the text:**

Line 204-219 : Descriptive data were presented in mean and standard deviations of variables. Pearson’s correlational analyses were conducted to examine bivariate relationships between psychological distress, social support, and coping strategies. After testing the assumptions, it was found that this data met the minimum criteria for conducting a moderating analysis using regression. Afterward, for testing potential moderator we used moderated regression model and carried out with the PROCESS analysis (v.3) with 5000 bootstrap samples and heteroscedasticity-consistent standard errors [38]. PROCESS is a conditional process modeling program that makes use of an ordinary least squares or logistic-based path analytical framework to test the moderation effect [39]. On the present study, social support is used as a moderator variable (W), psychological distress was the independent variable (X), and approach coping strategies (Y1) and avoidance coping strategies (Y2) were the dependent variable. Two separate model were examine to predicted that (1) Social support would increase the negative correlation between approach coping and psychological distress and (2) Social support would decrease the positive correlation between avoidance coping and psychological distress. The moderator effect for the two-interaction term indicates a significant moderator effect with p < 0.05 and 95% of level confidence interval (CI).

19. **Comments :**

*Main Manuscript – Results: Tables; never use vertical bars.*

**Our response:**

The tables have been revised.

**Changes made in the text:**

Line 229-273: Table 1 to Table 6

20. **Comments :**

*Main Manuscript – Results: Table 5; please report all statistical indices. Please check how you reported the lower interval of the CI.*

**Our response:**

We revised as suggested

21. **Comments :**

*Main Manuscript – Results: Table 6 is quite enigmatic. Please explain with more details.*

**Our response:**

We clarify the explanation for Table 6.

22. **Comments :**

*Main Manuscript – Discussion; compared to the extensive Introduction section, the Discussion section falls very short both by length and content. Please adjust.*

**Our response:**

We thank the reviewer for the constructive comments to improve this manuscript.

23. **Comments :**

*Main Manuscript – Conclusions: strictly taken, it’s not about the amount and availability of social support, but it’s about a person’s ability to perceive social support as beneficial.*

**Our response:**

We revised the conclusion as suggested.

**Changes made in the text:**

Line 326-328: Most importantly, it’s not about the amount and availability of social support, but it’s about mother’s ability to perceive social support as beneficial for her in this difficult situation.
