# Supplementary material for: Coping strategies and psychological distress among mothers during COVID-19 pandemic: The moderating role of social support
Source: PLoS One. 2024 Apr 2;19(4):e0300365. doi: 10.1371/journal.pone.0300365 (PMC10986941; doi:10.1371/journal.pone.0300365)
Supplement: Attachment — Submitted filename: plos one comments.docx [file pone.0300365.s004.docx]

The authors touch on a very important topic regarding social support, coping and psychological distress and highlighted the need to support mothers in the face of an overwhelming global crisis.

1. The first sentence of your second hypothesis seems to have been a repeat. Kindly check it
2. The last sentence of the “participants” section seems redundant as it is repeated in the next section
3. Under “data analysis" section, authors mentioned that “after testing the assumptions…”, please mention the specific assumptions tested here.
4. Under “data analysis”, please change the word “correlation” to “association” in the sentence that begins with “two separate models…..”
5. Can the authors provide justification why their regression models were not adjusted or controlled for confounders such as age, residence, etc?
6. The authors should also be cautious in their use of “correlated” when in fact they are reporting the results from a regression model. The appropriate word is “associated”.
7. The authors should be cautious with the use of words that connote causal relationships. For instance, in the conclusion section, the authors mention that social support decreases psychological distress. This statement is not factual because the study had a cross-sectional design. The authors should rather say, for instance, “social support is negatively associated with psychological distress”. Kindly check throughout the manuscript to correct these minor errors.
8. Lastly, there are a few grammatical errors that need to be checked to enhance the clarity of the manuscript. I encourage the authors to consider this.
