# Supplementary material for: Coping strategies and psychological distress among mothers during COVID-19 pandemic: The moderating role of social support
Source: PLoS One. 2024 Apr 2;19(4):e0300365. doi: 10.1371/journal.pone.0300365 (PMC10986941; doi:10.1371/journal.pone.0300365)
Supplement: Attachment — Submitted filename: plos review .docx [file pone.0300365.s005.docx]

**Reviewer Comments to the Author**

Dear PLOS One team of editorials, thank you for giving me the chance to review the manuscript entitled " Coping Strategies and psychological distress among mothers during COVID-19 Pandemic: the moderating role of social support”

This study gives very important results regarding coping strategies and psychological distress among mothers during the COVID-19 pandemic. However, in a few areas, here are my comments.

1. From the title to the end variation in COVID-19 writing. Better to prefer COVID-19 instead of Covid-19. please correct it
2. While reading the abstract your Respondents are not clear …Which software you used for data analysis is not clear ....it must be included in the abstract…. Make the abstract catchier and reader-friendly...background, methods, results, and conclusion must be clear.
3. In the social support section: your reviewed findings [29,30] were from non-COVID-19 time how could you relate to pandemic time both situations are different …
4. In your sample population mother of neonates, infants, toddlers, preschool, schools, and adolescents were included, and obviously there were lots of variations in the study population, and stress anxiety and depression levels might be affected according to the child’s age ……. how do you justify
5. What are the measures taken to control the confounders?
6. How do you get access to the mother’s network it must be explained. For data collection how do you access the online platform? how and where do you start to share tools first …
7. is convenience sampling a suitable sampling technique for your study ...What about networking or snowball sampling… as you explained your data collection type…please explain why convenience sampling?
8. In line 147: I think the convenience sampling method is for selecting a sample population and an online survey is used for your data collection. A questionnaire (structured or semi-structured, or unstructured) is your tool ...please make it clear
9. In procedure many places future tense is used and needs to be corrected
10. In methods you mention that your present study is part of a longitudinal project …. but in limitation line 318 you mention that the cross-sectional design of this study implies no causation effect can be determined…... So please specify your study design. In your methodology section, you must mention your study design, sampling technique, and research tool …. clearly for ease to the reader.
11. Use correct tense, grammar, sentence, spelling, paraphrase, consistency…etc needs correction
