# Supplementary material for: Coping strategies and psychological distress among mothers during COVID-19 pandemic: The moderating role of social support
Source: PLoS One. 2024 Apr 2;19(4):e0300365. doi: 10.1371/journal.pone.0300365 (PMC10986941; doi:10.1371/journal.pone.0300365)
Supplement: Attachment — Submitted filename: Response to Reviewers.docx [file pone.0300365.s006.docx]

Submission **Coping strategies and psychological distress among mothers during COVID-19 pandemic: the moderating role of social support**

Dear reviewer(s),

First, we would like to thank you for your constructive input and for recognizing our effort to improve this paper. Those comments genuinely boost our motivation to work better on our current study. Regarding to your feedback, below we have written down your comments, which we put in *italics*. Your comments are followed by our response and the associated changes made in the manuscript. You will find that we adapted the text in all cases. We hope that you will appreciate the new version and we look forward to finalizing this draft and preparing it for publication.

**Reviewer #2**

1. **Comment:**

*From the title to the end variation in COVID-19 writing. Better to prefer COVID-19 instead of Covid-19. please correct it.*

**Our response:**

Thank you for this remark. We have corrected the COVID-19 writing on the whole manuscript.

**Changes made in the text:**

Whole manuscript.

2. **Comment:**

*While reading the abstract your Respondents are not clear …Which software you used for data analysis is not clear ....it must be included in the abstract…. Make the abstract catchier and reader-friendly...background, methods, results, and conclusion must be clear.*

**Our response:**

Thank you for this suggestion, we incorporated them in the abstract page 2, especially on line 8-14 for the method explanation.

**Changes made in the text:**

Abstract page 2.

3. **Comment:**

*In the social support section: your reviewed findings [29,30] were from non-COVID-19 time how could you relate to pandemic time both situations are different …*

**Our response:**

Thank you for your valuable input. We added several findings that shows similar conclusion in COVID-19 time to support our arguments.

**Changes made in the text:**

We have added reference [30] in page 5-6 (line 87-88) highlighted in yellow to support our reviewed findings based on reference [29]. We have added an explanation that the findings in reference [1] were based from a study in the pandemic situation, this also supports our reviewed findings based on reference [31] (previously [30]; see page 5-6 line 93-95, highlighted in yellow).

4. **Comment:**

*In your sample population mother of neonates, infants, toddlers, preschool, schools, and adolescents were included, and obviously there were lots of variations in the study population, and stress anxiety and depression levels might be affected according to the child’s age ……. how do you justify*

**Our response:**

We agree with the suggestion(s). We did not collect data on the age of children of the respondents. We added this into the limitation of this study.

**Changes made in the text:**

End of discussion section, page 19 line 317-320.

5. **Comment:**

*What are the measures taken to control the confounders?*

**Our response:**

Thank you for raising this important point regarding control of the confounders in our study. We did not control the confounders and focused on relationship between the variables that we investigated.

**Changes made in the text:**

No changes have been made.

6. **Comment:**

*How do you get access to the mother’s network it must be explained. For data collection how do you access the online platform? how and where do you start to share tools first …*

**Our response:**

We elaborated the data collection procedures being used in this study.

**Changes made in the text:**

We added the explanation in the “procedures” section on page 8-9 line 154-163.

7. **Comment:**

*Is convenience sampling a suitable sampling technique for your study ...What about networking or snowball sampling… as you explained your data collection type…please explain why convenience sampling?*

**Our response:**

We used the term convenience sampling referring to Clark (2017): “*A convenience sample is a nonprobability sample that involves a group of elements that is easily accessible to a researcher.*”

Reference: Clark, R. (2017). Convenience sample. The Blackwell Encyclopedia of Sociology, 1–2. https://doi.org/10.1002/9781405165518.wbeosc131.pub2

**Changes made in the text:**

No changes have been made.

8. **Comment:**

*In line 147: I think the convenience sampling method is for selecting a sample population and an online survey is used for your data collection. A questionnaire (structured or semi-structured, or unstructured) is your tool ...please make it clear*

**Our response:**

Thank you for your valuable input, we incorporate your suggestion in ‘participants’ and ‘procedures’ section.

**Changes made in the text:**

Procedure and participants section page 8-9.

9. **Comment:**

*In procedure many places future tense is used and needs to be corrected*

**Our response:**

We thank the reviewer for the constructive comment. We have corrected the tenses in the procedures section.

**Changes made in the text:**

We corrected the tenses in procedure section on page 8-9.

10. **Comment:**

*In methods you mention that your present study is part of a longitudinal project …. but in limitation line 318 you mention that the cross-sectional design of this study implies no causation effect can be determined…... So please specify your study design. In your methodology section, you must mention your study design, sampling technique, and research tool …. clearly for ease to the reader.*

**Our response:**

The present study is part of a longitudinal project investigating the psychological distress of Indonesian mothers and its determinant factors during the Covid-19 pandemic. For the present report, only data from the first data collection time point was presented, using cross-sectional approach.

**Changes made in the text:**

We have provided a more detailed explanation of what we mean on methods section on page 8 line 131-144.

11. **Comment:**

*Use correct tense, grammar, sentence, spelling, paraphrase, consistency…etc needs correction*

**Our response:**

We have recheck all manuscript thoroughly.

**Changes made in the text:**

Whole manuscript.

**Reviewer #3**

1. **Comment:**

*The first sentence of your second hypothesis seems to have been a repeat. Kindly check it*

**Our response:**

Thank you, we have corrected the second hypothesis.

**Changes made in the text:**

Current study section, page 7 line 134-137.

2. **Comment:**

*The last sentence of the “participants” section seems redundant as it is repeated in the next section*

**Our response:**

Thank you for your input. We have modified the procedure section to avoid sentence repetition.

**Changes made in the text:**

Procedure section, page 8.

3. **Comment:**

*Under “data analysis" section, authors mentioned that “after testing the assumptions…”, please mention the specific assumptions tested here.*

**Our response:**

We have elaborate the assumption we tested, namely testing the potential moderator

**Changes made in the text:**

Data analysis section, page 11 line 213-220.

4. **Comment:**

*Under “data analysis”, please change the word “correlation” to “association” in the sentence that begins with “two separate models…..”*

**Our response:**

Thank you for your input. We have revised it.

**Changes made in the text:**

Data analysis section, page 11 line 217.

5. **Comment:**

*Can the authors provide justification why their regression models were not adjusted or controlled for confounders such as age, residence, etc?*

**Our response:**

Thank you for raising this important point regarding control of the confounders in our study. We did not control the confounders and focused on relationship between the variables that we investigated.

**Changes made in the text:**

No changes has been made.

6. **Comment:**

*The authors should also be cautious in their use of “correlated” when in fact they are reporting the results from a regression model. The appropriate word is “associated”.*

**Our response:**

We have replace the word correlation/correlated into associated/association in the entire manuscript.

**Changes made in the text:**

Conclusion section page 19 line 324, discussion section page 18 line 284 and line 300.

7. **Comment:**

*The authors should be cautious with the use of words that connote causal relationships. For instance, in the conclusion section, the authors mention that social support decreases psychological distress. This statement is not factual because the study had a cross-sectional design. The authors should rather say, for instance, “social support is negatively associated with psychological distress”. Kindly check throughout the manuscript to correct these minor errors.*

**Our response:**

We have check throughout the manuscript to correct the words that that connote causal relationships

**Changes made in the text:**

Discussion section page 18 line 287 and line 300

8. **Comment:**

*Lastly, there are a few grammatical errors that need to be checked to enhance the clarity of the manuscript. I encourage the authors to consider this.*

**Our response:**

We have recheck all manuscript thoroughly.

**Changes made in the text:**

Whole document.
