# Supplementary material for: Coping strategies and psychological distress among mothers during COVID-19 pandemic: The moderating role of social support
Source: PLoS One. 2024 Apr 2;19(4):e0300365. doi: 10.1371/journal.pone.0300365 (PMC10986941; doi:10.1371/journal.pone.0300365)
Supplement: Attachment — Submitted filename: Response to Reviewers.docx [file pone.0300365.s007.docx]

Submission **Coping strategies and psychological distress among mothers during COVID-19 pandemic: the moderating role of social support**

Dear reviewer(s),

First, we would like to thank you for your constructive input and for recognizing our effort to improve this paper. Regarding to your feedback, below we have written down your comments, which we put in *italics*. Your comments are followed by our response and the associated changes made in the manuscript. You will find that we adapted the text in all cases. We hope that you will appreciate the new version and we look forward to finalizing this draft and preparing it for publication.

**Reviewer #4**

1. **Comment:**

*Thank you for the opportunity provided peer review for this interesting article on “Coping strategies and psychological distress among mothers during COVID-19 pandemic: the moderating role of social support”. The article involved 1534 Indonesian mothers and it explores that moderate and high levels of social support moderated the relationship between approach coping strategies and psychological distress (B=.041, CI .007-.075). When the mother uses approach coping, her psychological distress will decrease further whenever she receives moderate and high level social support. Any level of social support moderated the relationship between avoidant coping and psychological distress (B=-.100, CI -.138--.061).*

*Online survey was used to collect data and what software was used to enter the data is it the IBM SPSS or other? If you used other data entry software to manage the data centrally then it’s better to indicate.*

**Our response:**

We thank the reviewer for the constructive comment. We have added the software being used in the research.

**Changes made in the text:**

We have added the software information in page 11 (line 220) highlighted in yellow: IBM SPSS 24 software was used to analyze the data.

.

2. **Comment:**

*Please provide a reference for the sentence found on line 45 “Among these effects of the COVID-19 pandemic on parenting, a specific and more profound influence had been found in maternal roles.” Since you choose mother as your study population, it needs to be well supported with concrete evidence as mothers are more exposed to stressors than male during the pandemic.*

**Our response:**

Thank you for your valuable input. We added references that shows mothers are more exposed to stressors than male during the pandemic to support our arguments.

**Changes made in the text:**

We have added reference [11] and [12] in page 3 (line 46) to support our arguments. We also adjust the references number following the additional.
